# Effects of Diaphragmatic Breathing in Primary Dysmenorrhea: A Randomized Controlled Trial

**DOI:** 10.1155/prm/5555669

**Published:** 2026-06-28

**Authors:** Seyda Toprak Celenay, Beyza Avci, Beyza Nur Pinarcik, Sila Celik

**Affiliations:** ^1^ Ankara Yildirim Beyazit University, Health Science Faculty, Department of Physiotherapy and Rehabilitation, Ankara, Türkiye, aybu.edu.tr; ^2^ Ankara Yildirim Beyazit University, Institute of Health Sciences, Physiotherapy and Rehabilitation Doctorate Program, Ankara, Türkiye, aybu.edu.tr

**Keywords:** breathing exercise, menstruation, pain, sleep, stress

## Abstract

**Objective:**

This study aimed to investigate the effects of diaphragmatic breathing (DB) on menstrual pain and symptoms, quality of life, stress level, and sleep quality in women with primary dysmenorrhea (PD).

**Methods:**

Women with PD were randomly divided into two groups: the DB (*n* = 22) and the control groups (*n* = 22). DB was performed 3 days a week during 2 menstrual periods. No treatment was performed in the control group. For the primary outcome, the pain intensity with the Visual Analog Scale (VAS); for secondary outcomes, menstrual symptoms with the Menstrual Symptom Questionnaire (MSQ); quality of life with the Dysmenorrhea Impact Scale–Revised Short Form (DIS‐R); stress level with the Perceived Stress Scale (PSS); and sleep quality with the Pittsburgh Sleep Quality Index (PSQI) were assessed pre‐ and postintervention.

**Results:**

In the postintervention period, there was a further decrease in VAS (*p* < 0.001), MSQ (*p* < 0.001), DIS‐R (*p* = 0.003), and PSS scores (*p* = 0.036) in the DB group; however, no difference in the PSQI score was found (*p* = 0.150). Moreover, there was a further decrease in MSQ scores in the DB group than in the control group (*p* = 0.044).

**Conclusion:**

DB may help reduce menstrual pain, symptoms, and stress and improve the quality of life in women with PD. Thus, DB can be used as a complementary method in addition to other therapies in PD management. However, due to factors such as the nonintervention control group, the use of subjective evaluation methods, and the presentation of short‐term results and per‐protocol analysis, the study’s findings should not be generalized and should be interpreted as modest between‐group effects.

**Trial Registration:** ClinicalTrials.gov identifier: NCT063212244

## 1. Introduction

Primary dysmenorrhea (PD) is defined as painful menstruation, affecting 50–90% of women. PD may be accompanied by some complaints such as low back pain, fatigue, nausea, vomiting, diarrhea, and insomnia [1]. Among these, women with moderate to severe symptoms reported disruption of daily activities, including school, work, social, and sports activities [2]. It has been indicated that excessive secretion of prostaglandins and decreased ovarian steroid hormone levels in the endometrium are causes of PD [1].

The treatments of PD include medications, such as nonsteroidal anti‐inflammatory drugs, oral contraceptive pills, and sedatives. However, the adverse effects of some medications, such as headache, indigestion, and drug dependency, lead to the patient’s lack of interest in receiving such treatments [3]. PD can be treated by using alternative and complementary therapies, including aromatherapy, massage, electrotherapy, and exercise [3–5].

Diaphragmatic breathing (DB), a type of breathing exercise, can also be used to reduce PD. DB provides relaxation and allows individuals to focus their minds and change their thoughts about their pain [6]. It increases parasympathetic activity and beta endorphin release and reduces pain perception and stress [7]. Moreover, the pelvic floor muscles relax while the diaphragm contracts during breathing, thus the DB can increase blood circulation in the pelvic region and reduce hypoxia and PD complaints [8]. There are limited studies related to breathing exercises in PD management [6, 7, 9], and it is seen that breathing exercises are usually applied together with different treatments (relaxation techniques, etc.), and these combined interventions have positive effects on menstrual pain [9]. Considering the high prevalence of PD and the negative effects of PD on psychological status, sleep, and quality of life [10], more studies are needed on the effects of DB in PD.

The current study aimed to investigate the effects of DB on menstrual pain and symptoms, quality of life, stress level, and sleep quality in women with PD.

## 2. Materials and Methods

### 2.1. Study Design and Participants

This randomized controlled study was approved by the local ethics committee of the university (Approval number: 10–499) and conducted in accordance with the Declaration of Helsinki. Written informed consent was obtained.

Women who applied to the physiotherapy and exercise counseling center, were 18 years of age or older, had been diagnosed with PD by a gynecologist, had PD symptoms according to PD Consensus Guidelines [11], had menstrual pain intensity of 4 cm or more according to the Visual Analog Scale (VAS) in the last 6 months, had regular menstrual cycles (28 ± 7 days), and volunteered to participate in the study were included. Exclusion criteria were pregnancy; oral contraceptive or antidepressant use; both copper and hormonal intrauterine device use; and/or history of other gynecological diseases and pulmonary, neurological, and/or systemic diseases; those who had abdominal or cardiothoracic surgery within the last year; and inability to attend at least 80% of the total sessions within the exercise program.

### 2.2. Outcome Measures

Physical and clinical data were recorded. Menstrual pain and symptoms, quality of life, stress level, and sleep quality were assessed with self‐reported questionnaires. Preintervention assessment was made on the first or second day of their menstruation. Postintervention assessment was performed on the first or second day of the next menstruation following the interventions in both groups.

### 2.3. Primary Outcome

The menstrual pain intensity was assessed with the VAS, consisting of a 10‐cm line. According to this scale, 0 means “no pain” and 10 means “very severe pain.” Participants were asked to mark the mean severity of pain they felt during their menstruation on the VAS. The marked point was measured with the help of a ruler, and the result obtained was recorded in cm [12].

### 2.4. Secondary Outcomes

The severity of menstrual symptoms was assessed with the Turkish version of the Menstrual Symptom Questionnaire (MSQ). The scale consists of 24 items. The scale is scored between 22 and 110, and the score of each item ranges from 1 (*never*) to 5 (*always*). A higher score indicates a higher severity of menstrual symptoms [13].

The effects of dysmenorrhea on quality of life were assessed with the Dysmenorrhea Impact Scale–Revised Short Form (DIS‐R), developed in Turkish by Gün et al. The scale consists of 13 items and is scored between 13 and 65. The score of each item ranges from 1 (*I disagree*) to 5 (*I completely agree*). A higher score indicates a decreased quality of life [14].

The stress level was assessed with the Turkish version of the Perceived Stress Scale (PSS), designed in 5‐scale Likert type. The scale consists of 14 items. It is scored between 0 and 56. The score of each item ranges from 0 (*never*) to 4 (*very often*). A higher score indicates that the individual has a higher perception of stress [15].

The sleep quality was assessed with the Turkish version of the Pittsburgh Sleep Quality Index (PSQI). The scale consists of 7 subscales: subjective sleep quality, sleep latency, sleep duration, habitual sleep activity, sleep disturbances, use of sleep medication, and daytime dysfunction. The total score from the scale is between 0 and 21, and a higher score indicates poor sleep quality [16].

### 2.5. Randomization

**FIGURE 1:**
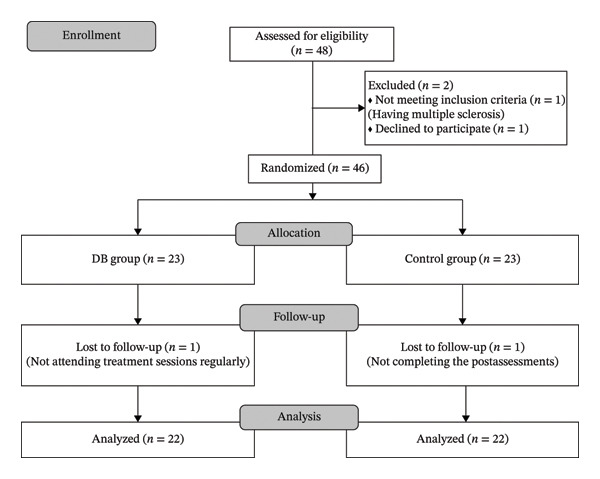
Flowchart of the participants.

Participants were randomized to the DB group or the control group by using an online block randomization list created with blocks of 4 [17]. Creating the randomization list and assigning participants to the interventions were conducted by a researcher who was not involved in the evaluation and intervention processes. Forty‐eight women with PD were assessed for eligibility. A total of 44 women (DB group = 22, control group = 22) completed the study. The flowchart of the participants is presented in Figure 1.

### 2.6. Intervention

**FIGURE 2:**
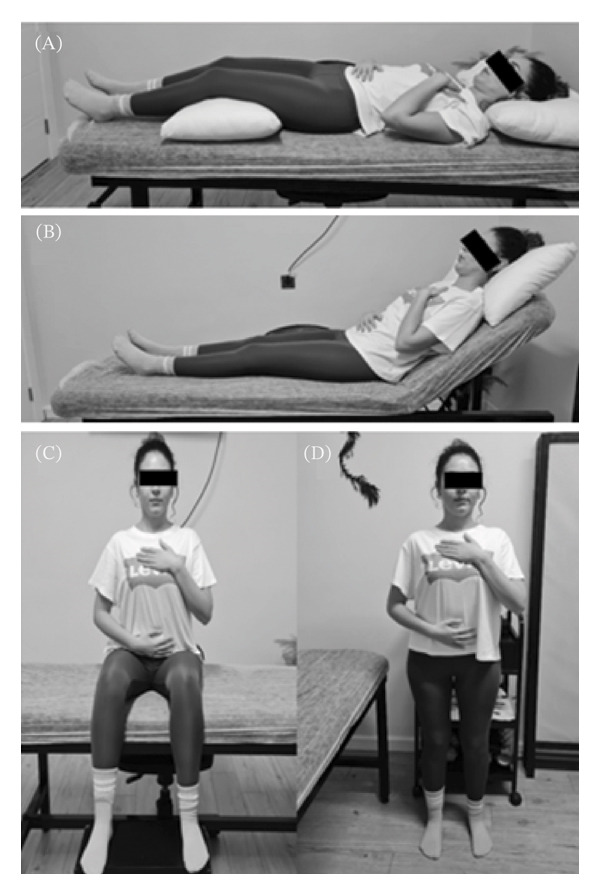
(A, B, C, D) Participants’ DB application positions.

The participants started the DB program after the first menstrual period when they were evaluated, and this program was applied as a home program 3 days a week for 2 menstrual periods. The DB group was first informed about the diaphragm muscle, its function, and the benefits of DB. A brochure related to this subject was prepared and given to the individuals. Then, the DB group was taught respiratory control and DB by a physiotherapist. During the DB, the participants placed one hand on the abdomen and the other on the chest. The participants were taught DB by focusing on the outward movement of the abdomen during inspiration and the inward movement of the abdomen during expiration. They were asked to inhale through the nose and exhale through the mouth. In the DB group, the participants applied DB in the supine and semirecumbent positions for a total of 10 min, 2 sets per day, during the first menstrual period (Figures 2A and B). DB was performed with 10 repetitions, and a 1‐min rest break was given. During the second menstrual period, DB was applied to individuals in sitting and standing positions for 3 sets per day for a total of 15 min (Figures 2C and D). The participants were instructed to record their DB programs on exercise checklists and submit them weekly to their physiotherapists. Moreover, individuals’ DB program follow‐up was checked by weekly online platforms/phone calls. As a result, the participants’ commitment to their exercises was also ensured.

The control group was not subjected to any intervention throughout the study and was asked to continue their daily routines. At the end of the study, the DB program was recommended to individuals in the control group.

### 2.7. Sample Size and Statistical Analysis

The G^∗^Power program was used to determine the sample size. First of all, a pilot study was conducted with 10 women with PD. Based on the VAS, MSQ, DIS‐R, PSS, and PSQI total scores, which are the outcome measures of this pilot study, the effect sizes were calculated as 0.505, 0.559, 0.503, 0.549, and 0.475, respectively. Accordingly, for the study power to be 80%, it was predicted that at least 18, 15, 18, 16, and 20 individuals would be included per group, respectively. According to the primary outcome (VAS score) of the study, it was determined that there should be at least 18 participants per group (36 participants in total) for the *α* = 0.05 and *β* = 0.20. It was calculated that a total of at least 44 participants should be recruited with a predicted data loss of 20%.

Number (*n*) and percentage (%) values were used to show the distribution of individuals in demographic information. The conformity of continuous variables to the normal distribution was evaluated graphically and with the Shapiro–Wilk test. Mean ± standard deviation and median (minimum–maximum) values were used to show descriptive statistics. In comparing VAS, MSQ, DIS‐R, PSS, and PSQI scores according to DB and control groups, an independent sample *t*‐test was used for parameters showing normal distribution, and a Mann–Whitney *U* test was used for parameters not showing normal distribution. In order to examine whether the parameters included in the study differed at the measurement times (pre‐ and postintervention), the dependent sample *t*‐test was used for parameters showing normal distribution, and the Wilcoxon signed rank test was used for parameters not showing normal distribution. Number (*n*), percentage (%), and chi‐square test statistics were given for the comparison of categorical variables.

Two‐way mixed ANOVA results were used to examine whether the parameter values for the two groups (exercise and control) differed at different measurement times (pretreatment and posttreatment). In cases where the ANOVA assumptions were not met, the nparLD *R* package (F1‐LD‐F1 design) was used. Effect size between the groups was expressed as partial eta squared (*η*2_p_), and values were interpreted as small (0.01), medium (0.06), and large (0.14) effects. Moreover, the effect size (*r*) was calculated for the changes observed during the treatment process in each group, and the *r* value was interpreted as small if it was between 0.10 and 0.29, medium if it was between 0.30 and 0.49, and large if it was ≥ 0.50 [18]. IBM SPSS Statistics 21.0 was used for analyses. The statistical significance level was accepted as *p* < 0.05.

## 3. Results

Individuals participated in the DB program regularly. Individuals did not use regular medication for PD during the study. No adverse events were reported throughout the study. No difference was found in terms of the characteristics of the individuals according to the DB group or control group (*p* > 0.05) (Table 1).

**TABLE 1:** Comparison of characteristics of groups.

Parameters	DB group (*n* = 22)		Control group (*n* = 22)		Test statistics	
	Mean ± SD	Median (min–max)	Mean ± SD	Median (min–max)	t; z	*p*
Age (years)	28.77 ± 5.05	27.5 (21–35)	26.23 ± 5.09	26.0 (19–35)	*z* = 1.845	0.065
BMI (kg/m^2^)	24.74 ± 4.77	23.6 (19.0–33.3)	22.29 ± 3.64	22.5 (16.3–30.1)	*z* = 1.726	0.084
Menarche (year)	12.95 ± 1.53	13.0 (10–15)	12.64 ± 1.53	12.5 (10–16)	*t* = 0.691	0.494
Menstrual cycle (days)	27.36 ± 4.25	28.0 (21–35)	27.59 ± 3.30	28.5 (21–31)	*z* = 0.384	0.701
	*n*	%	*n*	%		
Smoking					—	
No	19	86.4	20	90.9		0.500^a^
Yes	3	13.6	2	9.1		
Alcohol consumption					—	
No	22	100.0	20	90.9		0.244^a^
Yes	0	0	2	0.1		

*Note: t*, independent sample *t*‐test; *z*, Mann–Whitney *U* test.

Abbreviations: BMI, body mass index; kg, kilogram; Max, maximum; Min, minimum; m^2^, meter square; *n*, number; SD, standard deviation; %, percent.

^a^Fisher’s exact value is given.

There was no significant difference between the VAS scores in the pre‐ and postintervention of the groups. There was no significant difference between the two time‐dependent measurements (pre‐ and postintervention) of the VAS scores in the control group (*p* > 0.05, *r* = 0.059). There was a significant difference between the two time‐dependent measurements (pre‐ and postintervention) of the VAS scores in the DB group (*p* < 0.001). The effect size of this difference seen in the DB group was determined to be large (*r* = 0.627). A significant group × time interaction was found in VAS scores (*η*2_p_ = 0.384, *p* < 0.001) (Table 2).

**TABLE 2:** Comparison of VAS, MSQ, DIS‐R, PSS, and PSQI scores within and between groups at pre‐ and postintervention.

Parameters	DB group (*n* = 22)		Control group (*n* = 22)		Test statistics			
	Mean ± SD [95% CI]	Median (min–max)	Mean ± SD [95% CI]	Median (min–max)	P (group)		p (GTI)	($\eta_{p}^{2}$)

VAS								
Preintervention	7.36 ± 2.10 [6.43, 8.29]	7.5 (4–10)	6.40 ± 1.66 [5.66, 7.14]	6.4 (4–9)	*z* = 1.635	0.102	< 0.001	0.384
Postintervention	5.27 ± 2.33 [4.24, 6.30]	5.5 (2–9)	6.36 ± 1.84 [5.54, 7.18]	6.0 (2–9)	*t* = 1.722	0.092		
p (Time)	*z* = 3.690; *p* < 0.001^∗^		*t* = 0.272; *p* = 0.789					
r	0.627		0.059					
MSQ								
Preintervention	76.32 ± 12.58 [70.74, 81.90]	76.5 (51–96)	71.86 ± 12.29 [66.41, 77.31]	73.5 (49–95)	*t* = 1.188	0.242	< 0.001	0.301
Postintervention	63.36 ± 13.69 [57.29, 69.43]	60.5 (31–93)	72.09 ± 14.22 [65.78, 78.40]	74.0 (39–96)	*t* = 2.073	0.044^∗^		
p (Time)	*t* = 4.674; *p* < 0.001^∗^		*t* = 0.164; *p* = 0.871					
r	0.714		0.036					
DIS‐R								
Preintervention	53.18 ± 7.00 [50.08, 56.28]	52.5 (40–65)	49.68 ± 6.56 [46.77, 52.59]	50.0 (38–62)	*t* = 1.711	0.095	0.001	0.246
Postintervention	45.64 ± 10.93 [40.79, 50.49]	48.0 (20–64)	50.73 ± 6.35 [47.91, 53.55]	50.5 (39–63)	*t* = 1.888	0.068		
p (Time)	*t* = 3.345; *p* = 0.003^∗^		*t* = 1.949; *p* = 0.065					
r	0.589		0.391					
PSS								
Preintervention	31.86 ± 7.94 [28.34, 35.38]	32.0 (16–52)	30.59 ± 8.05 [27.02, 34.16]	30.0 (15–52)	*t* = 0.528	0.600	0.027	0.111
Postintervention	28.00 ± 7.93 [24.48, 31.52]	29.0 (13–40)	31.23 ± 9.20 [27.15, 35.31]	29.5 (16–52)	*t* = 1.246	0.220		
p (Time)	*t* = 2.235; *p* = 0.036^∗^		*t* = 0.678; *p* = 0.505					
r	0.438		0.146					
PSQI								
Preintervention	7.09 ± 3.28 [5.64, 8.54]	7.0 (2–14)	6.09 ± 2.37 [5.04, 7.14]	6.0 (2–11)	*t* = 1.160	0.253	0.099	0.063
Postintervention	6.23 ± 3.28 [4.78, 7.68]	6.0 (0–12)	6.41 ± 2.06 [5.50, 7.32]	6.0 (4–11)	*t* = 0.220	0.827		
p (Time)	*t* = 1.493; *p* = 0.150		*t* = 0.802; *p* = 0.432					
r	0.309		0.172					

*Note:* DIS‐R, Dysmenorrhea Impact Scale–Revised Short Form; *t*, independent sample *t*‐test; *z*, Mann–Whitney *U* test; *t*, dependent sample *t*‐test; *z*, Wilcoxon signed rank test; *η*2_p_, partial eta squared; *r*, effect size.

Abbreviations: CI, confidence interval; GTI, group × time interaction; Max, maximum; Min, minimum; MSQ, Menstrual Symptom Questionnaire; PSQI, Pittsburgh Sleep Quality Index; PSS, Perceived Stress Scale; SD, standard deviation; VAS, visual analog scale.

^∗^ *p* < 0.05.

There was no significant difference between the MSQ scores in the pre‐ and postintervention of the groups. There was no significant difference between the two time‐dependent measurements (pre‐ and postintervention) of the MSQ scores in the control group (*p* > 0.05, *r* = 0.036). There was a significant difference between the two time‐dependent measurements (pre‐ and postintervention) of the MSQ scores in the DB group (*p* < 0.001). The mean MSQ score after treatment in the DB group decreased compared to before treatment. The effect size of this difference seen in the DB group was determined to be large (*r* = 0.714). A significant difference was found between the posttreatment MSQ scores of the DB and control groups (*p* = 0.044). A significant group × time interaction was found in MSQ scores (*η*2_p_ = 0.301, *p* < 0.001) (Table 2).

No significant difference was found between the DIS‐R scores in the pre‐ and postintervention of the groups. No significant difference was found between the two time‐dependent measurements (pre‐ and postintervention) of the DIS‐R scores in the control group (*p* > 0.05, *r* = 0.391). A significant difference was found between the two time‐dependent measurements (pre‐ and postintervention) of the DIS‐R scores in the DB group (*p* = 0.003). The mean DIS‐R score after treatment in the DB group decreased compared to pretreatment. The effect size of this difference seen in the DB group was determined to be large (*r* = 0.589). A significant group × time interaction was found in DIS‐R scores (*η*2_p_ = 0.246, *p* = 0.001) (Table 2).

No significant difference was found between the PSS scores in the pre‐ and postintervention of the groups. There was no significant difference between the two time‐dependent measurements (pre‐ and postintervention) of PSS scores in the control group (*p* > 0.05, *r* = 0.146). There was a significant difference between the two time‐dependent measurements (pre‐ and postintervention) of PSS scores in the DB group (*p* = 0.036). The mean PSS score after treatment in the DB group decreased compared to pretreatment. The effect size of this difference seen in the DB group was determined to be medium (*r* = 0.438). A significant group × time interaction was found in PSS scores (*η*2_p_ = 0.111, *p* = 0.027) (Table 2).

There was no significant difference between the PSQI scores in the pre‐ and postintervention of the groups. In addition, there was no significant difference between the two time‐dependent measurements (pre‐ and postintervention) of PSQI scores in both the control and DB groups (*p* > 0.05, control group *r* = 0.172, DB group *r* = 0.309). No significant group × time interaction was found in PSQI scores (*η*2_p_ = 0.063, *p* > 0.05) (Table 2).

No statistically significant differences were found between the subdimensions (subjective sleep quality, sleep latency, sleep duration, habitual sleep activity, sleep disturbances, use of sleep medication, and daytime dysfunction) of the PSQI according to the groups (*p* > 0.05). When changes over time were examined, no statistically significant differences were found between the exercise and control groups. The group × time interaction was not found to be significant in the PSQI subdimension scores, and it was determined that the effects of the exercise and control groups on the PSQI subdimension scores were similar (*p* > 0.05). The effect sizes of subjective sleep quality, sleep latency, sleep duration, habitual sleep efficiency, sleep disturbances, use of sleep medication and daytime dysfunction within the DB group were calculated as *r* = 0.167, *r* = 0.109, *r* = 0.356, *r* = 0.082, *r* = 0.124, *r* = 0.213, and *r* = 0.117, respectively. For the control group, the within‐group effect sizes for the same parameters were *r* = 0.213, *r* = 0.0002, *r* = 0.072, *r* = 0.206, *r* = 0.281, *r* = 0.213, and *r* = 0.390, respectively. Furthermore, no significant group × time interaction was found for these variables, with effect sizes and *p* values determined as (*η*2_p_ = 0.030, *p* = 0.261), (*η*2_p_ = 0.003, *p* = 0.722), (*η*2_p_ = 0.036, *p* = 0.215), (*η*2_p_ = 0.010, *p* = 0.518), (*η*2_p_ = 0.033, *p* = 0.235), (*η*2_p_ = 0.001, *p* = 0.999), and (*η*2_p_ = 0.065, *p* = 0.096), respectively (Table 3).

**TABLE 3:** PSQI subdimensions scores within and between groups at pre‐ and postintervention.

Parameters	DB group (*n* = 22)		Control group (*n* = 22)		Test statistics			
	Mean ± SD [95% CI]	Median (min–max)	Mean ± SD [95% CI]	Median (min–max)	P (group)		p (GTI)	(*η*2_p_)
Subjective sleep quality								
Preintervention	1.50 ± 0.74 [1.17, 1.83]	1.0 (1–3)	1.09 ± 0.53 [0.85, 1.33]	1.0 (0–2)	*z* = 1.829	0.067	0.261	0.030
Postintervention	1.36 ± 0.85 [0.98, 1.74]	1.5 (0–3)	1.18 ± 0.59 [0.92, 1.44]	1.0 (0–2)	*z* = 0.947	0.344		
p (Time)	*z* = 0.775; *p* = 0.439		*z* = 1.000; *p* = 0.317					
r	0.167		0.213					
Sleep latency								
Preintervention	1.41 ± 1.18 [0.89, 1.93]	1.5 (0–3)	1.27 ± 0.70 [0.96, 1.58]	1.0 (0–3)	*z* = 0.357	0.721	0.722	0.003
Postintervention	1.32 ± 1.21 [0.78, 1.86]	1.0 (0–3)	1.27 ± 0.70 [0.96, 1.58]	1.0 (0–2)	*z* = 0.012	0.990		
p (Time)	*z* = 0.504; *p* = 0.614		*z* = 0.001; *p* = 0.999					
r	0.109		0.0002					
Sleep duration								
Preintervention	0.59 ± 0,73 [0.27, 0.91]	0.0 (0–2)	0.59 ± 0.73 [0.27, 0.91]	0.0 (0–2)	*z* = 0.001	0.999	0.215	0.036
Postintervention	0.27 ± 0,70 [‐0.04, 0.58]	0.0 (0–2)	0.55 ± 0.74 [0.22, 0.88]	0.0 (0–2)	*z* = 1.712	0.087		
p (Time)	*z* = 1.748; *p* = 0.080		*z* = 0.333; *p* = 0.739					
r	0.356		0.072					
Habitual sleep efficiency								
Preintervention	0.41 ± 0.91 [0.01, 0.81]	0.0 (0–3)	0.36 ± 0.66 [0.07, 0.65]	0.0 (0–2)	*z* = 0.248	0.804	0.518	0.010
Postintervention	0.36 ± 0.66 [0.07, 0.65]	0.0 (0–2)	0.18 ± 0.66 [‐0.11, 0.47]	0.0 (0–3)	*z* = 1.468	0.142		
p (Time)	*z* = 0.378; *p* = 0.705		*z* = 0.966; *p* = 0.334					
r	0.082		0.206					
Sleep disturbances								
Preintervention	1.59 ± 0.67 [1.29, 1.89]	1.5 (1–3)	1.55 ± 0.74 [1.22, 1.88]	1.5 (0–3)	*z* = 0.142	0.887	0.235	0.033
Postintervention	1.50 ± 0.86 [1.12, 1.88]	2.0 (0–3)	1.68 ± 0.78 [1.33, 2.03]	2.0 (0–3)	*z* = 0.618	0.536		
p (Time)	*z* = 0.577; *p* = 0.564		*z* = 1.342; *p* = 0.180					
r	0.124		0.281					
Sleeping medication								
Preintervention	0.09 ± 0.43 [‐0.10, 0.28]	0.0 (0–2)	0.14 ± 0.47 [‐0.07, 0.35]	0.0 (0–2)	*z* = 0.564	0.573	0.999	0.001
Postintervention	0.0 ± 0.0 [0.00, 0.00]	0.0 (0–0)	0.05 ± 0.21 [‐0.04, 0.14]	0.0 (0–1)	*z* = 1.000	0.317		
p (Time)	*z* = 1.000; *p* = 0.317		*z* = 1.000; *p* = 0.317					
r	0.213		0.213					
Daytime dysfunction								
Preintervention	1.50 ± 0.91 [1.10, 1.90]	1.5 (0–3)	1.14 ± 0.89 [0.75, 1.53]	1.0 (0–3)	*z* = 1.272	0.203	0.096	0.065
Postintervention	1.41 ± 0.85 [1.03, 1.79]	1.0 (0–3)	1.45 ± 0.86 [1.07, 1.83]	1.0 (0–3)	*z* = 0.218	0.828		
p (time)	*z* = 0.540; *p* = 0.589		*z* = 1.941; *p* = 0.052					
r	0.117		0.390					

*Note:* Mann–Whitney *U* test, *z*, Wilcoxon signed rank test; *η*2_p_, partial eta squared; *r*, effect size.

Abbreviations: CI, confidence interval; GTI, group × time interaction; Max, maximum; Min, minimum; SD, standard deviation.

## 4. Discussion

This study suggests that DB may be beneficial in reducing menstrual pain, symptoms, and stress levels and improving quality of life in women with PD. No statistically positive effect on sleep was detected. In the intergroup comparison, DB was found to provide greater improvement in menstrual pain, symptoms, quality of life, and stress compared to the control group and had large clinical significance for these parameters. Only the sleep parameter showed moderate clinical significance.

In a study conducted by Choudhury et al. [19], it was shown that parasympathetic activity decreases, sympathetic activity increases, and thus pain perception increases during the luteal and follicular phases of the menstrual cycle. Pal et al. [20] reported that breathing exercises increase the activity of the parasympathetic system and reported that slow breathing improves autonomic function compared to fast breathing. In a study conducted by Purnamasari et al. [21] on adolescent individuals, the acute effect of deep breathing exercises performed for 30 min was examined, and it was observed that the severity of pain caused by PD decreased. Wahyuni et al. [6] reported that relaxation techniques together with deep breathing were effective in reducing the level of pain in university students with PD. Our study was also planned as a randomized controlled trial, investigating the effects of DB in the management of PD. In our study, DB was found to be effective in reducing menstrual pain intensity. No change in menstrual pain was observed in the control group. The clinical significance value for the menstrual pain was greater in the DB group than in the control group. These results may be due to the effects of DB, such as a decrease in abnormal sympathetic activity and pain perception and an increase in relaxation [22]. Moreover, for pelvic pain, a 10 mm–15 mm reduction on a 100 mm VAS is generally recognized as the minimal clinically important difference (MCID) [23]. In our study, we also observed a decrease of approximately 20 mm in the VAS score related to menstrual pain in the DB group. Thus, DB may be useful in the management of PD.

The most common symptom of PD is pain, and additional menstrual symptoms (low back pain, fatigue, nausea, vomiting, insomnia, etc.) may accompany it. Ganesh et al. [24] reported a statistically insignificant decrease in menstrual symptoms in both groups in which they applied fast and slow breathing exercises. Bulbuli et al. [7] reported that breathing exercises were effective in improving menstrual symptoms in individuals with PD. In our study, in the DB group, a decrease in menstrual symptoms was observed. No difference in menstrual symptoms was found in the control group. These symptoms improved more in the DB group compared to the control group. Moreover, the clinical significance value for the menstrual symptoms was greater in the DB group than in the control group. It may be clinically important to consider these results in PD management.

It has been reported that DB, which is generally applied in addition to relaxation training, reduces pain in PD and increases the quality of life [25]. In our study, only DB was applied to women with PD, and as a result of the study, the DB was seen to improve quality of life and was of great clinical importance. These results may be due to decreasing menstrual pain intensity, symptoms, and stress levels. Thus, DB can be considered as a complementary treatment method in PD. In contrast to this, no difference in quality of life was found in the control group.

Menstruation is a physiological period that includes many biopsychosocial components. It has been reported that there may be a bidirectional relationship between psychological disorders and PD [10]. Lee and Kim [26] examined the effects of stress and self‐esteem on PD and showed that stress directly affects the frequency and severity of menstrual symptoms and indirectly affects the severity of pain. A systematic review shows that DB can reduce both physiological and psychological stress by affecting blood pressure, respiration, and cortisol levels [27]. In our study, similar to the literature data, DB was found to be effective in reducing the perceived stress level. These results may be due to the increase in relaxation and parasympathetic activity with DB. However, no change in perceived stress level was found in the control group. The clinical significance value for the stress level was greater in the DB group than in the control group.

Pain is a factor that affects sleep quality by shortening REM duration and total sleep duration [28]. Aktaş reported that 14.9% of women with PD experience insomnia during menstruation [29]. No study has been found examining the effect of DB on sleep quality in women with PD. Kirmizigil and Demiralp [9] applied combined exercises (stretching, strengthening, and pelvic floor exercises) with DB for 8 weeks in PD management and reported that sleep quality improved at the end of the study. In this study, it was found that DB alone was not statistically significant in improving sleep quality but had a moderate effect from a clinical perspective. More detailed studies are needed on this subject.

There were some limitations of our study. The use of self‐reported measurements instead of objective measurements may also be a limitation of this study. Nevertheless, since self‐report outcome measures reflect the subjective nature of pain, it is accepted as the gold standard in evaluating pain outcomes [30]. Due to the nature of the applications and self‐reported outcomes, the assessors or the participants could not be blinded in this study. It may be a source of bias in the results of the study. Moreover, we designed a nonintervention control group to observe the natural process of menstrual symptoms in individuals. The lack of intervention in the control group may have led to expectation and placebo bias. Further studies on this topic should consider randomized sham‐controlled designs, as this will strengthen the validity of these data. Another limitation may also be the short follow‐up period. By revealing the effects in the longer term, the most optimal DB application parameters, such as application frequency and duration, should be determined in order to maintain the effect in the long term. Finally, although the study was designed as a randomized controlled trial, the analyses were performed on participants who completed the study (per‐protocol analysis, which may overestimate effects). Participants who dropped out of follow‐up were not included in the analyses. The absence of an intention‐to‐treat analysis is one of the potential limitations of the study.

According to the results, this study provides preliminary evidence that DB has been shown to help reduce menstrual pain, symptoms, and stress and improve quality of life. According to these results, DB can be used as a complementary method in addition to other therapies in PD management. However, due to factors such as the nonintervention control group, the use of subjective evaluation methods, and the presentation of short‐term results and per‐protocol analysis, the study’s findings should not be generalized and should be interpreted as modest between‐group effects. Further studies with high methodological quality are needed on this topic.

## Author Contributions

CRediT: Seyda Toprak Celenay: project administration, conceptualization, formal analysis, investigation, methodology, resources, writing–original draft, and writing–review and editing. Beyza Avci: conceptualization, data curation, formal analysis, investigation, methodology, resources, writing–original draft, and writing–review and editing. Beyza Nur Pinarcik: conceptualization, data curation, formal analysis, investigation, methodology, resources, writing–original draft, and writing–review and editing. Sila Celik: conceptualization, data curation, formal analysis, investigation, methodology, resources, writing–original draft, and writing–review and editing.

## Funding

This research did not receive any specific grant from funding agencies in the public, commercial, or not‐for‐profit sectors.

## Conflicts of Interest

The authors declare no conflicts of interest.

## Data Availability

The data that support the findings of this study are available on request from the corresponding author.
